# Comparative evaluation of image registration methods with different interest regions in lung cancer radiotherapy

**DOI:** 10.1186/s12880-019-0402-9

**Published:** 2019-12-26

**Authors:** Xiaohui Cao, Ming Liu, Fushan Zhai, Nan Li, Feng Li, Chaoen Bao, Yinliang Liu, Gang Chen

**Affiliations:** 1 Department of Radiotherapy and Oncology, The Third Hospital of Hebei Medical University, Shijiazhuang, 050017 Hebei China; 2 Department of Respiratory Medicine, The Third Hospital of Hebei Medical University, No.139 Ziqiang Road,Qiaoxi district, Shijiazhuang, 050017 Hebei China

**Keywords:** Lung cancer, Cone beam computed tomography, Accuracy, Position deviation, Time consumption

## Abstract

**Background:**

Lung cancer is a leading cause of morbidity and mortality worldwide. Radiotherapy for lung cancer is beneficial in both the radical and palliative settings, and technologic advances in recent years now afford an opportunity for this treatment to be more targeted than ever before. Although the delivery of more accurate forms of radiotherapy has minimized the risks of side-effects, how to utilize this treatment to optimize outcomes remains questionable. This study aimed to evaluate the accuracy of cone beam computed tomography (CBCT) image registration used in image-guided radiotherapy, providing reasonable guidance for clinic application of CBCT in lung cancer.

**Methods:**

A total of 53 patients with lung carcinoma including 34 central and 19 peripheral lesions were collected in this study. Varian-IX linear accelerator on-board imaging (OBI) system was used to acquire CBCT scans in three-dimensional (3D) conformal radiotherapy before delivery. Different regions (whole lung/target/vertebrae/ipsilateral structure) were manually registered, and the position deviation and the registration time were analyzed.

**Results:**

It was suggested that 34 cases belonged to central type and 19 cases belonged to peripheral type. The volume of left lung and right lung was 1242.98 ± 452.46 cc, 1689.69 ± 574.31 cc, respectively. Tumor size was 6.65 ± 3.87 cm in diameter, and 129.67 ± 136.48 cc in volume. The percentage of left lung and right lung was 6.17 ± 1.24%, 4.74 ± 0.38%, respectively. The position deviation value and absolute value of image registration methods of X, Y and Z axis were not significant (*P* > 0.05). However, registration time (s) between whole lung registration group, tumor registration group, vertebral body registration group, affected lung registration group, and artificial registration group, was 3.651 ± 0.867 s, 1.144 ± 0.129 s, 1.226 ± 0.126 s, 2.081 ± 0.427 s, 179.491 ± 71.975 s, respectively. The differences were significant (*P* < 0.05). The registration differences between small tumor group and large tumor group were not statistically significant (*P* > 0.05).

**Conclusion:**

The automatic image matching of OBI is accuracy and high reliability in recognition of offset error. Registering body or ipsilateral structure is recommended to be used in CBCT for lung cancer.

## Background

Lung cancer with high incidence and mortality rate is the first malignant tumors in China, which is induced by interaction between genetic and environmental factors [1]. Radiotherapy is an important treatment method for lung cancer [2, 3]. With the increasing application of intensity modulated radiotherapy, the treatment location requires more and more high accuracy. For instance, the accuracy of target delineation has been improved by the use of magnetic resonance imaging (MRI), positron emission tomography-computed tomography (CT), and four-dimensional CT [4]. Furthermore, localization of tumor on the treatment delivery machine can be detected using megavoltage portal imaging, kilovoltage (kV) planar imaging, cone-beam CT (CBCT), and stereoscopic imaging [5]. Among these radiotherapy methods, CBCT provides three-dimensional (3D) or four-dimensional (4D) reconstructed images for improving patient position accuracy [6]. Notably, CBCT has been widely used to detect prostate cancer [7], breast cancer [8], primary oesophageal cancer [9], cervical cancer [10] and colon cancer [11]. 4D CBCT of the lung is proved to be an effective tool for motion management in radiotherapy but presents a challenge because of slow gantry rotation times [12]. Simpson et al. (2010) suggested that use of image-guided radiotherapy (IGRT) technology is increasing and growing further [13]. Full potential of advanced technology such as intensity-modulated radiation therapy (IMRT) can be exploited better if it is combined with IGRT [14, 15]. However, there are few researches on the automatic matching accuracy of x-ray volume on-board imaging (OBI) in IGRT system.

In this study, the accuracy, advantages and disadvantages of different registration sites were compared by CBCT images and planned CT images in 53 patients with lung cancer, in order to provide reference for clinical application of CBCT.

## Methods

### Study design

From October 2017 to May 2018, all 53 cases diagonozed with lung cancer patients were collected in Third Hospital of Herbei Medical Universitiy, Shijiazhuang, China. All patients took supine position with head cushion B pillow and both hands embracing the head, and were fixed with thermoplastic body membrane. Chest CT scanning ranged from the cricothyroid membrane to the lower edge of the diaphragm was done using Siemens Somotom-sensation Plus-16 spiral CT scanner, and the scanned images were transmitted to the Computerized Medical Systems (CMS) treatment planning system. Among 53 patients, 34 cases belonged to central type and 19 cases belonged to peripheral type.

Inclusion criteria: Histological or cytological diagnosis of lung cancer; having no self-reported history of any malignant tumor; willing and able to give written informed consent; and no active or chronic infection with human immunodeficiency virus (HIV), hepatitis B, or hepatitis C.

Exclusion criteria: received any chemotherapy or radiotherapy prior to surgery; pregnant or breast feeding patients; a history or presence of other malignancy; and clinically significant autoimmune disease.

### CBCT image acquisition

The OBI linear accelerator of Warian IX was used as the IGRT system to acquire onboard CBCT images before patient treatment delivery. The image acquisition parameters were 120 kV, 528 mA, clockwise gantry rotation from 180° to 180°, fast scan, gantry speed 360°/min, collimator cassette M20, F1 filter, and 330 frames. CBCT scans were performed for the first time in routine for patients, and then 1–2 times a week. The CBCT images scanned by each patient during the first treatment were taken as the object of study and studied offline.

### Comparison of OBI automatic matching area and method setting

Most scholars believe that gray-level registration with shift deviation less than 0.1 mm has higher accuracy [4], so this study used gray-level registration. Four automatic registration methods including whole lung (all lung within the scope of CBCT scanning), tumors [(1 cm in the 3D direction of gross tumor volume (GTV)], vertebral body (whole vertebral body within the scope of CBCT scanning in the 3D direction) and affected lung (affected lung tissues within the scope of CBCT scanning) and artificial registration method were selected for off-line registration of CT-CBCT images. The location of the target center of patients and the deviation values of the target center position of the scratch images in the left and right, up and down, front and back directions were recorded, separately. The time-consuming and accuracy differences between 5 different registration methods were analyzed.

### Statistical analysis

The statistical analysis was performed on different registration methods using the one-way analysis of variance (ANOVA) followed by Post hoc analysis using two independent sample t-test using statistical product and service solutions (SPSS) software (SPSS V.24.0, IBM, IL, USA). Data were expressed as mean ± standard deviation (SD). *P* < 0.05 was considered as a statistically significant difference.

### Results

General information of the patients was shown in Table 1. It was suggested that a total of 53 patients (32 males and 21 females) at age of 69 ± 10.3 years old with lung tumors showing the symptoms such as coughing, pain in the chest area, wheezing, bone pain, headache and tumors diagnosed with CT were enrolled in this study. The cases included 33 left lung cases, 20 right lung cases. Furthermore, 34 cases belonged to central type and 19 cases belonged to peripheral type. Left lung volume was 1242.98 ± 452.46 cc, and right lung volume was 1689.69 ± 574.31 cc. Tumor size was 6.65 ± 3.87 cm in diameter, and 129.67 ± 136.48 cc in volume. The percentage of left lung and right lung was 6.17 ± 1.24%, 4.74 ± 0.38%, respectively.

**Table 1:** General information

General information	Data
Number of patients	*N* = 53
Age (Median, Range)	69 (40–83)
Sex (Male, %)	32 (60%)
Left Lung volume (cc)	1242.98 ± 452.46
Right Lung volume (cc)	1689.69 ± 574.31
Tumor location	
Left Lung	33
Right Lung	20
Tumor genotyping	
Central type	34
Peripheral type	19
Tumor size	
Diameter (cm)	6.65 ± 3.87
Volume (cc)	129.67 ± 136.48
Volume (cc)	80.28 ± 6.82
Lung tumor percentage	
Left lung tumor [tumor volume/(left lung tumor + left lung volume)]	6.17 ± 1.24%
Right lung tumor [tumor volume/(right lung tumor + right lung volume)]	4.74 ± 0.38%

A total of 265 sets of registration data were collected from 53 patients, and the deviation and absolute values of image registration methods of different parts on X, Y and Z axis were demonstrated in Table 2. Statistical analysis showed that the position deviation values were X axis (mm): 0.226 ± 2.900, 0.623 ± 3.295, 0.453 ± 2.866, 0.000 ± 3.322, 0.377 ± 2.669; Y axis (mm): 0.396 ± 7.292, 0.208 ± 5.055, 0.094 ± 5.838, 0.491 ± 6.116, 0.094 ± 5.274; Z axis (mm): 1.981 ± 2.678, 1.830 ± 2.847, 1.302 ± 2.334, 2.000 ± 2.908, 1.264 ± 2.543, respectively. The difference of X, Y and Z axis was not significant (*P* > 0.05). In contrast, registration times were significantly different for whole lung registration (3.651 ± 0.867 s), tumor registration (1.144 ± 0.129 s), vertebral body registration (1.226 ± 0.126 s), affected lung registration (2.081 ± 0.427) and artificial registration (*P* < 0.05).

**Table 2:** Deviation value, registration time and statistical value at different registration sites on X, Y or Z axis (x ± s)

Index	Whole lung	Tumor	Vertebral body	Affected lung	Artificial registration	F	P
Registration time (s)	3.651 ± 0.867	1.144 ± 0.129	1.226 ± 0.126	2.081 ± 0.427	179.491 ± 71.975	322.209	0.000
Deviation value							
X axis (mm)	0.226 ± 2.900	0.623 ± 3.295	0.453 ± 2.866	0.000 ± 3.322	0.377 ± 2.669	0.323	0.863
Y axis (mm)	0.396 ± 7.292	0.208 ± 5.055	0.094 ± 5.838	0.491 ± 6.116	0.094 ± 5.274	0.110	0.979
Z axis (mm)	1.981 ± 2.678	1.830 ± 2.847	1.302 ± 2.334	2.000 ± 2.908	1.264 ± 2.543	0.988	0.415
Absolute value							
X axis (mm)	2.189 ± 1.892	2.396 ± 2.323	2.302 ± 1.739	2.453 ± 2.215	2.038 ± 1.743	0.368	0.831
Y axis (mm)	5.340 ± 4.926	3.906 ± 3.170	4.472 ± 3.703	4.642 ± 3.962	4.057 ± 3.325	1.129	0.343
Z axis (mm)	2.623 ± 2.040	2.698 ± 2.025	2.057 ± 1.692	2.792 ± 2.143	2.094 ± 1.904	1.678	0.155

In addition, all 53 patients were divided into two groups including smaller tumor group and large tumor group according to the median tumor volume of 80.28 ± 6.82 cc, and the registration differences between the two groups under different registration methods were compared, respectively. Statistical analysis showed that the differences were not statistically significant (*P* > 0.05) (Table 3).

**Table 3:** Comparison of general conditions and different registration methods between small tumors and large tumors

Index	Small tumors (*n* = 27)	Large tumors (*n* = 26)	P
Age (Years)	72 ± 11.3	67 ± 13.2	0.222
Sex (Male, %)	15 (56%)	17 (65%)	0.474
Left Lung volume (cc)	1087.29 ± 292.41	1404.65 ± 532.47	0.009
Right Lung volume (cc)	1547.73 ± 527.97	1837.12 ± 593.11	0.066
Tumor location			0.917
Left Lung	17	16	
Right Lung	10	10	
Tumor genotyping			0.058
Central type	14	20	
Peripheral type	13	6	
Whole lung registration (absolute value)			
X axis (mm)	2.148 ± 1.634	2.231 ± 2.160	0.876
Y axis (mm)	4.741 ± 4.129	5.962 ± 5.653	0.372
Z axis (mm)	2.630 ± 2.272	2.615 ± 1.813	0.980
Tumor registration (absolute value)			
X axis (mm)	2.593 ± 1.824	2.192 ± 2.772	0.291
Y axis (mm)	4.037 ± 2.981	3.769 ± 3.410	0.762
Z axis (mm)	2.407 ± 1.907	3.000 ± 2.135	0.536
Vertebral registration (absolute value)			
X axis (mm)	2.704 ± 1.857	1.885 ± 1.532	0.086
Y axis (mm)	4.593 ± 3.856	4.346 ± 3.611	0.811
Z axis (mm)	1.889 ± 1.476	2.231 ± 1.904	0.467
Affected lung registration (absolute value)			
X axis (mm)	2.407 ± 1.986	2.500 ± 2.470	0.939
Y axis (mm)	4.815 ± 4.114	4.462 ± 3.870	0.749
Z axis (mm)	2.815 ± 1.618	2.769 ± 2.612	0.881
Artificial registration (absolute value)			
X axis (mm)	2.259 ± 1.831	1.808 ± 1.650	0.350
Y axis (mm)	4.296 ± 3.698	3.808 ± 2.940	0.598
Z axis (mm)	1.852 ± 1.586	2.346 ± 2.190	0.351

### Discussion

Image guided radiotherapy (IGRT) provides the basis for precise treatment of intensity-modulated radiotherapy, and kV X-ray imaging is used frequently during a course of radiation therapy to improve the precision and accuracy of the delivery of the treatment of lung cancer [16]. IGRT can verify the treatment position prior to the implementation of the treatment, measure and analyze the three-dimensional error of the central position of tumors and correct it online, which has become one of the important bases for the implementation of precision therapy [17]. In the system of IGRT, CBCT has attracted significant attention from practitioners who seek to enhance diagnosis and treatment for their patients because it provides multidimensional and dimensionally accurate images for diagnosis and treatment planning [18] However, CBCT is a conical beam scanning, and has a certain gap between fan Beam CT in the image clarity, especially for lung tumors with obvious respiration problems [19]. Therefore, how to quickly and accurately register CBCT images with planned images, especially for chest tumors that are easily affected by respiration, needs a further investigation. Lung cancer is the leading cause of cancer deaths worldwide especially in developing countries [20]. In this study, we evaluated the accuracy of CBCT image registration used in image-guided radiotherapy of lung cancer.

Image registration with tumor as marker point was first put into CBCT clinical application in a previous study. If there is a significant difference in density between tumor and surrounding lung tissue, the tumor itself has the basic conditions as a registration marker [21]. However, due to the long time it takes to obtain CBCT in practice, the tumor will move with breathing [22], and the obtained CBCT image includes information about breathing movement, thus the accuracy of image matching is obviously affected. Moreover, with the treatment, the tumor is likely to shrink, and shows obvious difference with planned image of the tumor, leading to the difficulty of registration [23]. In the present study, anatomical structures such as vertebral body, protrusion, etc. were used because they are significantly different compared to the density of the surrounding tissues and do not change significantly with the passage of treatment. The analysis of 160 sets of registration data of 8 patients with lung cancer using different registration methods suggested that thoracic vertebrae can be used for the image guidance of lung cancer images guided radiotherapy [24]. The study of CBCT image registration in 25 patients with lung cancer found that the results of central type and peripheral type registration are different. For central type of lung cancer, protrusion registration is the best, but the spine registration is the worst. For peripheral type of lung cancer, tumor registration mark is the best, and the spine, protrusion registration is the worst [25]. In another study, 15 cases of lung cancer patients with different anatomical region registration methods were compared by the planned CT planning tumor volume (PTV) and CBCT GTV. It was concluded that the accuracy of bone registration in the same registration range is worse than that of grayscale registration [26]. Ottosson et al. (2010) proved that the pendulum error measured by different registration methods is different and requires different out-of-the-place boundaries. Manual registration is manually regulated by doctors to be exactly the same as the planned CT image, which is considered to be the most accurate registration method, but takes a long time and affects the work efficiency [27]. In this study, compare to automatic registration, the results showed that there are no statistical differences in the direction of x-axis, y-axis and z-axis, regardless of the selection of the whole lung, tumor, vertebral body, or the affected lung in the manual registration. We speculate the reasons are that in the registration process, doctors also refer to different anatomical signs, and give only more comprehensive consideration. If the tumor boundary is clear, it will be first confirmed that the tumor registration is consistent. If the tumor is closely related to the endangered organ, such as the spinal cord, the vertebral body may be used as a reference sign for a larger proportion. Therefore, there are no statistical differences between manual registration and automatic registration of different parts.

Nakamura et al. (2015) demonstrated that the accuracies of correlation models derived using the shortest modeling period of 20 s are almost identical to those obtained over the longest modeling period of 40 s [28]. In terms of registration time, previous study found that the smaller the registration range, the less information needs to be integrated, and the less time takes [29]. The registration time for tumor group and the vertebral body group is more than 1 s, the affected lung group is more than 2 s, the whole lung group is nearly 4 s, and the artificial registration group is more than 3 min, and the differences are obvious. Therefore, it is confirmed that if the scanned image is not consistent with the planned image, it will take a long time for registration. In the study, it is also found that the smaller the registration range, the more accurate the organization registration, the less care for the outside of the registration frame. According to the median number of tumor volume 80.28 ± 6.82 cc, the patients were divided into small tumor group and large tumor group. Comparing the general conditions of the two groups, it is found that the difference of left lung volume is statistically significant. The error numerical sizes of different registration methods are also compared, and the differences are not statistically significant. However, in the study, it is suggested that the peripheral small tumors with large breathing amplitude are significantly different from other registration methods indicating that we need to increase the samples for a further study.

## Conclusion

In summary, the automatic matching function of OBI system is precise in chest application, accurate in the identification of offset error and high in reliability. This method shows advantages such as the smaller registration range, the more accurate organization registration, and the less care for the outside of the registration frame, but has disadvantages such as small selection of registration areas, and large deviation value for peripheral lung cancer. Considering the registration time and other factors, it is recommended to choose the affected lung or whole lung as the registration area. However, there are still limitations. First, the cases involved in this study are limited, so individual difference has a great influence on the data. In addition, through comprehensive analysis, the tumor in the course of treatment may be reduced, and some tumors and surrounding tissues do not have obvious density differences, resulting in registration difficulties.

## Data Availability

The datasets used and/or analyzed during the current study are available from the corresponding author on reasonable request.

## Abbreviations

3D: Three-dimensional
4D: Four-dimensional
ANOVA: One-way analysis of variance
CBCT: Cone beam computed tomography
CMS: Computerized medical systems
CT: Computed tomography
GTV: Gross tumor volume
HIV: Human immunodeficiency virus
IGRT: Image-guided radiotherapy
IMRT: Intensity-modulated radiation therapy
kV: Kilovoltage
MRI: Magnetic resonance imaging
OBI: On-board imaging
PTV: Planning tumor volume
SD: Standard deviation
SPSS: Statistical product and service solutions
